# EUS‐Guided Versus Percutaneous Transhepatic Drainage of Liver Abscesses: A Multicenter Endohepatology Study in Western Japan (EPIC‐LA Study)

**DOI:** 10.1111/den.70067

**Published:** 2025-11-27

**Authors:** Takeshi Ogura, Taira Kuroda, Takanori Matsuura, Jun Kitadai, Koh Kitagawa, Masahiro Itonaga, Kotaro Takeshita, Tomoaki Matsumori, Tomoya Emori, Mamoru Takenaka, Hajime Imai, Koichiro Mandai, Shuhei Shintani, Nao Fujimori, Hideyuki Shiomi, Masanori Asada, Ryota Sagami, Hirotsugu Maruyama, Tsukasa Ikeura, Masaaki Shimatani, Hidefumi Nishikiori, Kazuyuki Matsumoto, Masahito Kokubu, Hideki Kamada, Yusuke Ishida, Akitoshi Hakoda, Masayuki Kitano

**Affiliations:** ^1^ Pancreatobiliary Advanced Medical Center Osaka Medical and Pharmaceutical University Hospital Osaka Japan; ^2^ Endoscopy Center Osaka Medical and Pharmaceutical University Hospital Osaka Japan; ^3^ 2nd Department of Internal Medicine Osaka Medical and Pharmaceutical University Osaka Japan; ^4^ Gastroenterology Center Ehime Prefectural Hospital Matsuyama Japan; ^5^ Division of Gastroenterology, Department of Internal Medicine Kobe University Graduate School of Medicine Kobe Japan; ^6^ Department of Gastroenterology Nara Medical University Kashihara Japan; ^7^ Second Department of Internal Medicine Wakayama Medical University Wakayama Japan; ^8^ Department of Gastroenterology Tane General Hospital Osaka Japan; ^9^ Department of Gastroenterology and Hepatology Kyoto University Graduate School of Medicine Kyoto Japan; ^10^ Department of Gastroenterology Wakayama Rosai Hospital Wakayama Japan; ^11^ Department of Gastroenterology and Hepatology Kindai University Faculty of Medicine Graduate School of Medical Sciences Osakasayama Japan; ^12^ Department of Gastroenterology Okanami General Hospital Iga Japan; ^13^ Department of Gastroenterology Kyoto Second Red Cross Hospital Kyoto Japan; ^14^ Department of Gastroenterology Shiga University of Medical Science Otsu Japan; ^15^ Department of Medicine and Bioregulatory Science, Graduate School of Medical Sciences Kyushu University Fukuoka Japan; ^16^ Division of Hepatobiliary and Pancreatic Diseases, Department of Gastroenterology Hyogo Medical University Hyogo Japan; ^17^ Department of Gastroenterology and Hepatology Japanese Red Cross Osaka Hospital Osaka Japan; ^18^ Department of Gastroenterology, Faculty of Medicine Oita University Oita Japan; ^19^ Department of Gastroenterology, Graduate School of Medicine Osaka Metropolitan University Osaka Japan; ^20^ Division of Gastroenterology and Hepatology Kansai Medical University Hospital Hirakata Japan; ^21^ Department of Gastroenterology and Hepatology Kansai Medical University Medical Center Moriguchi Japan; ^22^ Department of Gastroenterology Oita San‐ai Medical Center Oita Japan; ^23^ Department of Endoscopy Okayama University Hospital Okayama Japan; ^24^ Department of Gastroenterology and Metabology Ehime University Graduate School of Medicine To‐on Japan; ^25^ Department of Gastroenterology and Neurology, Faculty of Medicine Kagawa University, Kagawa Japan; ^26^ Department of Gastroenterology and Medicine, Faculty of Medicine Fukuoka University Fukuoka Japan

**Keywords:** drainage, endoscopic ultrasound‐guided liver abscess drainage, EUS, liver abscess, percutaneous transhepatic liver abscess drainage

## Abstract

**Objective:**

Percutaneous transhepatic liver abscess drainage (PTAD) and endoscopic ultrasound‐guided liver abscess drainage (EUS‐LAD) have several limitations. Recently, because of technical improvements in echoendoscope maneuvers, EUS‐guided access for the right hepatic lobe has been reported. The aim of this multicenter, retrospective study was to compare clinical outcomes of PTAD and EUS‐LAD including the right hepatic lobe in West Japan.

**Method:**

This retrospective, multicenter study included consecutive patients with liver abscesses between January 2019 and November 2024. The primary outcome in this study was the clinical success rate compared between EUS‐LAD and PTAD.

**Results:**

During the study period, 1012 consecutive patients developed liver abscesses. Of them, 734 patients were excluded, 43 underwent EUS‐LAD and 235 patients underwent PTAD. After propensity score‐matched analysis, the clinical success rate was significantly higher in the EUS‐LAD group (97.7%, 42/43) than in the PTAD group (79.1%, 34/43) (*p* = 0.007). After a propensity score‐matched analysis, 25 patients were included in each group. The clinical success rate was significantly higher in the EUS‐LAD group (100%, 25/25) than in the PTAD group (84%, 21/25) (*p* = 0.037). Adverse events were also significantly higher in the PTAD group (16%, 5/25) than in the EUS‐LAD group (*p* = 0.025). In addition, the median length of hospital stay was significantly shorter in the EUS‐LAD group (15 days) than in the PTAD group (22 days) (*p* = 0.005).

**Conclusions:**

EUS‐LAD using a metal stent might be one of the options, but further randomized, controlled trials are needed.

## Graphical abstract

The aim of this multicenter, retrospective study was to compare clinical outcomes of percutaneous transhepatic liver abscess drainage (PTAD) and endoscopic ultrasound‐guided liver abscess drainage (EUS‐LAD). After a propensity score‐matched analysis, the clinical success rate was significantly higher in the EUS‐LAD group, and adverse events were significantly higher in the PTAD group. In addition, the median length of hospital stay was significantly shorter in the EUS‐LAD group. Therefore, EUS‐LAD using a metal stent might be one of the options.

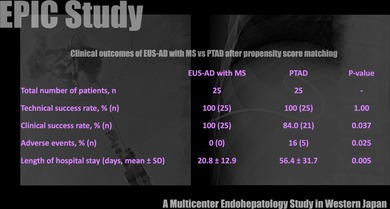

## 1 Introduction

Liver abscesses are common in clinical practice. The majority of patients with liver abscesses can be treated conservatively. Liver abscess drainage is indicated for cases involving the left lobe, a thin rim of hepatic parenchyma (< 10 mm), impending rupture on imaging, or nonresponse to conservative treatment after 3–5 days [1]. The gold standard technique for liver abscess drainage is the percutaneous transhepatic approach [2], but to overcome several disadvantages of percutaneous transhepatic liver abscess drainage (PTAD), such as external drainage or the risk of self‐tube removal, endoscopic ultrasound‐guided liver abscess drainage (EUS‐LAD) has been reported [3, 4, 5, 6]. EUS‐LAD may have several benefits, such as internal drainage, being able to avoid small vessel injury by using an elevator or high‐quality imaging, or the high drainage effect when EUS‐LAD is performed using a metal stent. Although this technique has recently been compared with PTAD, there have been several limitations, such as including a small number of patients and a case of left hepatic liver abscess drainage [7]. Recently, because of technical improvements in echoendoscope maneuvers, EUS‐guided access for the right hepatic lobe has been reported [8, 9, 10, 11]. Therefore, further evaluation of the clinical impact of EUS‐LAD is needed. The aim of this multicenter, retrospective study was to compare clinical outcomes of PTAD and EUS‐LAD in West Japan.

## 2 Patients and Method

This retrospective, multicenter study included consecutive patients with liver abscesses between January 2019 and November 2024. In this study, indications for EUS‐LAD were as follows: (1) abscess with a thin rim of hepatic parenchyma (< 10 mm) around it; (2) size of liver abscess > 50 mm; or (3) nonresponse to medical therapy after 3–5 days [1, 2]. The exclusion criteria were as follows: inaccessible duodenum, such as surgically altered anatomy; massive ascites around the puncture route by EUS and the percutaneous transhepatic approach; risk of significant bleeding; complicating multilocular liver abscess except oligolocular liver abscess; and combination with other drainage techniques such as PTAD.

### 2.1 Technical Tips for Drainage Under EUS and the Percutaneous Transhepatic Approach

**FIGURE 1:**
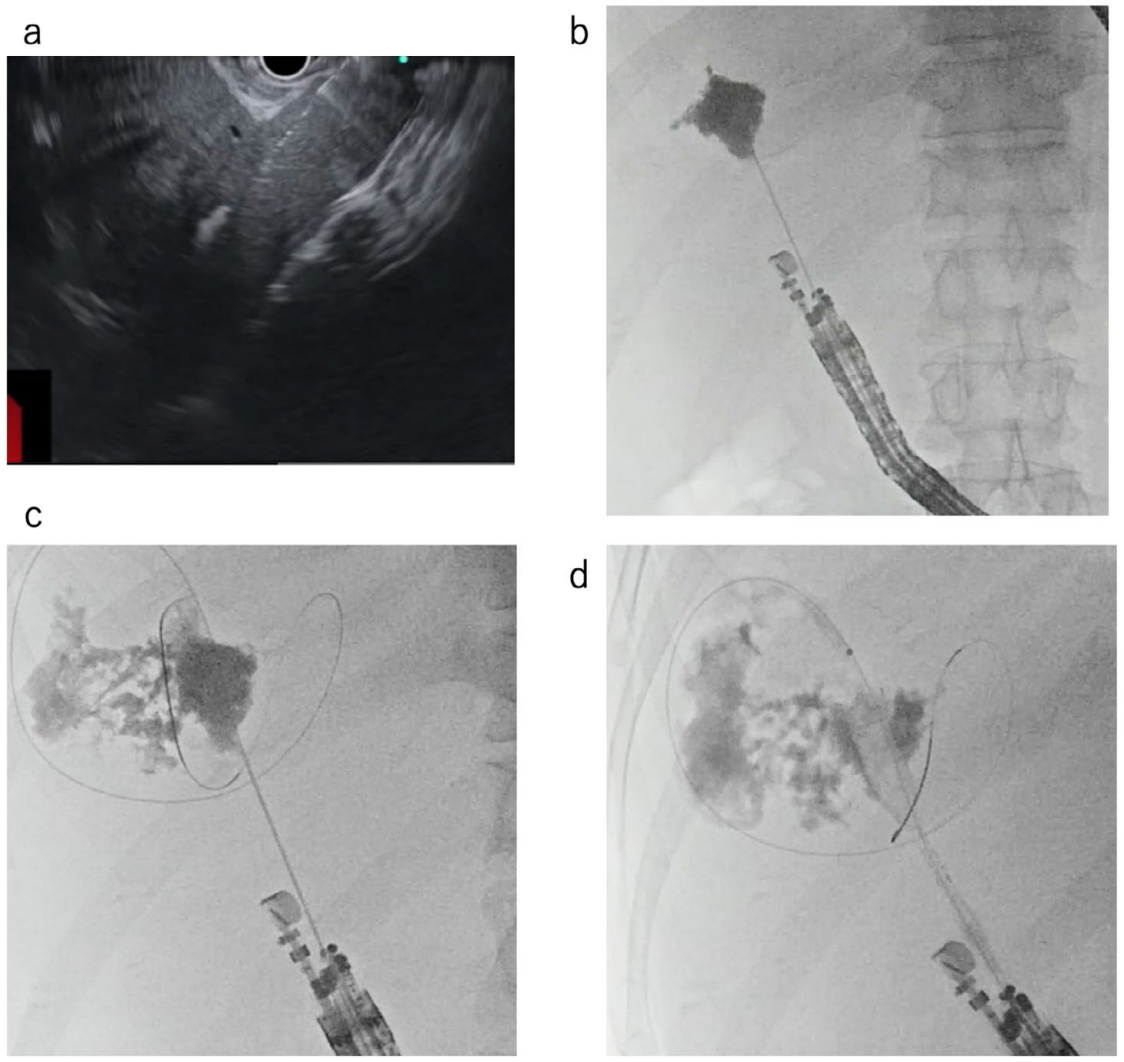
Technical tips for endoscopic ultrasound‐guided liver abscess drainage. (a) The liver abscess is punctured using a 19‐G needle. (b) The contrast medium is injected into the liver abscess. (c) A 0.025‐inch guidewire is deployed within the liver abscess. (d) Partially covered self‐expandable metal stent is placed from the liver abscess to the stomach or duodenum.

Figure 1 shows the EUS‐LAD technique. In cases of EUS‐LAD for the right hepatic lobe, the echoendoscope maneuver was performed under fluoroscopic guidance to prevent duodenal perforation, as previously described [10]. Next, the liver abscess was punctured using a 19‐G needle (EZ Shot 3 Plus, Olympus, Tokyo, Japan) (Figure 1a), and the fluid contents were aspirated. After the contrast medium injection (Figure 1b) and 0.025‐inch guidewire (VisiGlide, Olympus; J‐Wire, JMIT, Shiga, Japan) deployment within the liver abscess (Figure 1c), the liver abscess and stomach wall were dilated using a 4‐mm balloon catheter (REN biliary dilation catheter; KANEKA, Osaka, Japan) or mechanical dilator (ES dilator; Zeon Medical, Tokyo, Japan), and a stent 8 mm or 10 mm diameter, length of 6, 8, 10, or 12 cm, partially covered self‐expandable metal stent (PCSEMS) (Niti‐S, and Spring Stopper; Taewoong Medical, Seoul, Korea), fully covered SEMS (FCSEMS) (HANAROSTENT Biliary Full Cover Benefit; M.I. Tech, Seoul, Korea) (BONA Biliary Full Cover Stent; Standard Sci Tech, Seoul, South Korea), or plastic stent (PS) (SUZAKU (KANEKA), Advanix J (Boston Scientific, Marlborough, MA, USA), QuickPlace (Olympus), and Flexima (Boston Scientific)), or endoscopic nasal biliary drainage tube (ENBD, 7‐Fr or 8.5‐Fr) was placed from the liver abscess to the stomach or duodenum (Figure 1d). In the present study, since the diameter of the liver abscess was over 10 cm, PCSEMS was basically selected. Furthermore, because the distal end of the metal stent was placed near the liver abscess wall, FCSEMS was chosen to prevent mucosal hyperplasia. If the liver abscess was large, drainage tube obstruction can be frequently complicated because the amounts of contents are high. Furthermore, the pressure of the contents of the liver abscess might be high. To prevent leakage, SEMS was selected. If the distance from the stomach or intestine to the liver abscess was far, stent migration into the abdominal cavity can be complicated. In such cases, ENBD tube deployment was considered. After EUS‐LAD, patients underwent computed tomography (CT) the day after EUS‐LAD to evaluate adverse events such as stent dislocation or migration. As a clinical follow‐up protocol, trans‐abdominal ultrasound or CT was performed at 7, 14, and 28 days to evaluate liver abscess size. After achieving clinical success, stent removal was attempted.

**FIGURE 2:**
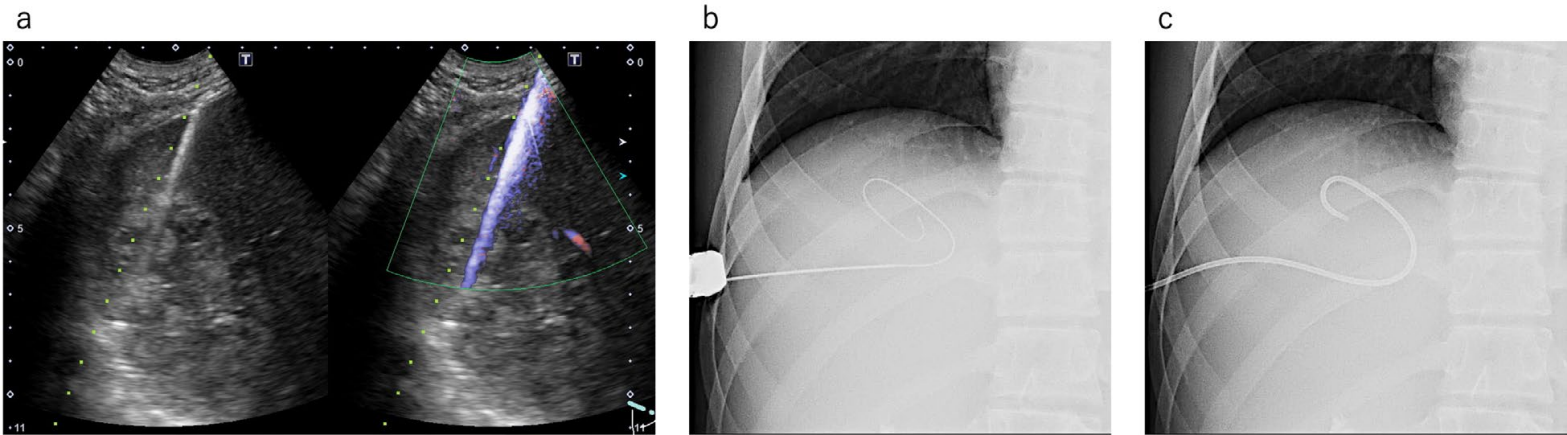
Technical tips for percutaneous transhepatic liver abscess drainage. (a) The liver abscess was punctured using an 18‐G aspiration needle under ultrasound guidance. (b) A 0.035‐inch guidewire is inserted into the cavity of the liver abscess. (c) A pigtail drainage tube is placed within the liver abscess.

Figure 2 shows the PTAD technique. The liver abscess was punctured using an 18‐G aspiration needle under ultrasound guidance (Figure 2a), and necrotic material was aspirated. The contrast medium was injected, and a 0.035‐inch guidewire was inserted into the cavity of the liver abscess (Figure 2b). Finally, a pigtail drainage tube (6.5, 7, 8, 10, or 12 Fr) was placed within the liver abscess (Figure 2c) and sutured to the skin. After PTAD tube deployment, scheduled washing was attempted on every day. As clinical follow‐up protocol, PTAD tube was discontinued once drainage output decreases, follow‐up CT was performed to evaluate liver abscess size. If clinical success was obtained, PTAD tube was removed. If the clinical effect was insufficient, the drainage tube size was increased.

### 2.2 Definitions and Statistical Analysis

The primary outcome in this study was the clinical success rate compared between EUS‐LAD and PTAD. Clinical success was defined as decreasing levels of inflammation on blood examinations or decreased size of the maximum liver abscess within 14 days after each procedure to 50% compared with that before EUS‐LAD or PTAD, and complete resolution of clinical symptoms, such as abdominal pain and fever. The technical success rate, adverse events, and length of hospital stay were evaluated as secondary outcomes. Technical success was defined as successful stent or tube deployment. Adverse events associated with EUS‐LAD were evaluated according to the severity grading system of the American Society for Gastrointestinal Endoscopy lexicon [12]. Duration of hospital stay was measured from the day of EUS‐LAD or PTAD to discharge. The diameter of the liver abscess was measured by CT. Procedure time was measured from liver abscess puncture to stent or external tube deployment. Liver abscess recurrence after stent removal in the EUS‐LAD group or tube removal in the PTAD group was defined as the typical symptoms such as fever, or abdominal pain with observation of liver abscess at the same site based on CT. The follow‐up period was measured from the day of performing EUS‐LAD to the final observation.

The Mann–Whitney *U*‐test and Fisher's exact test were used to compare patients' characteristics and laboratory data between the two groups. Propensity score matching was performed using Cox proportional hazards regression analysis to create a propensity score with a logistic regression model. One‐to‐one matching without replacement was performed with a caliper width of 0.2, and the resulting score‐matched pairs were used in subsequent analyses. Descriptive statistics are presented as mean ± standard deviation (SD) values or median and interquartile range for continuous variables and as frequencies for categorical variables. Differences with a *p*‐value less than 0.05 were considered significant. All data were statistically analyzed using SPSS version 13.0 statistical software (SPSS, Chicago, IL).

## 3 Results

**FIGURE 3:**
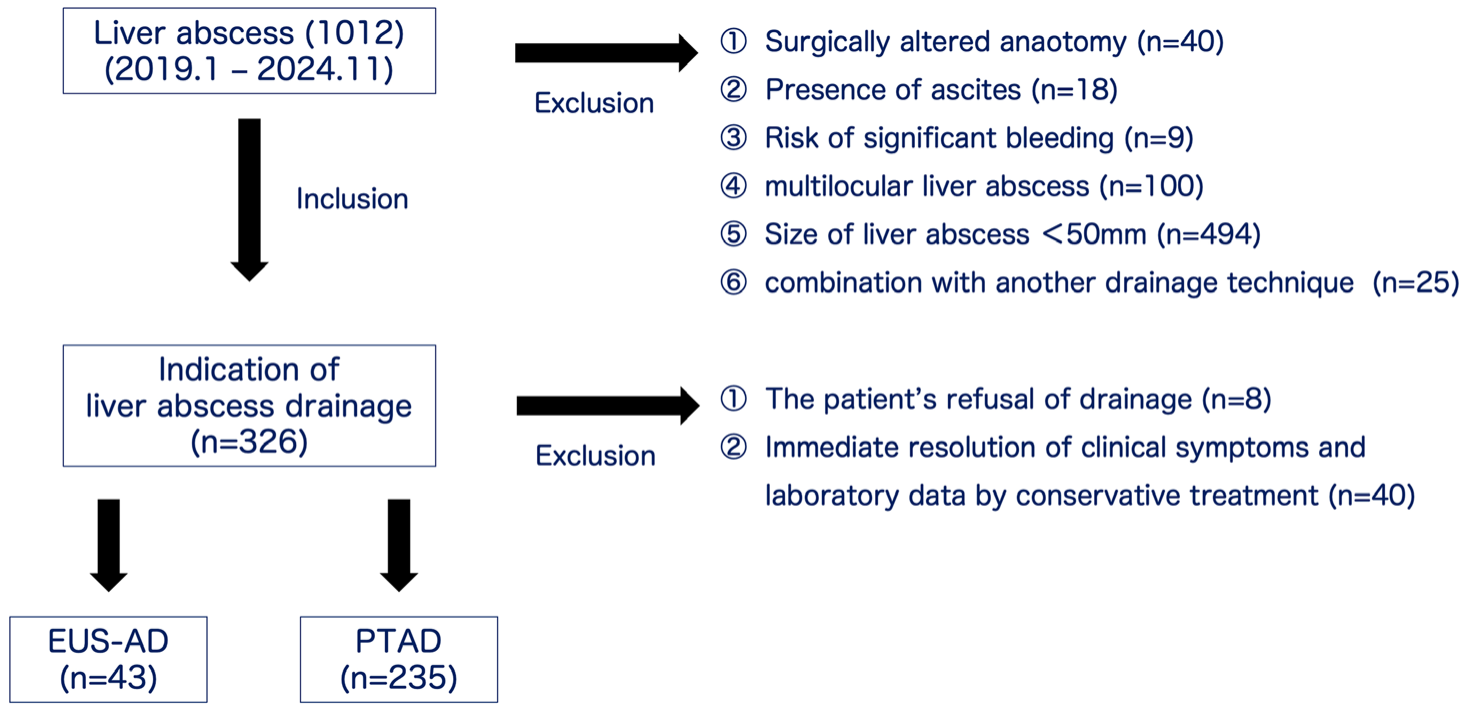
Patient's flow chart in the present study.

During the study period, 1012 consecutive patients developed liver abscesses. Of them, a total of 283 patients were enrolled in this study (Figure 3).

Table 1 shows the patients' characteristics. Of the 283 patients, 43 (25 men; median age, 71 years) underwent EUS‐LAD, and 235 patients (160 men; median age, 76 years) underwent PTAD. The mean Charlson Comorbidity Index (CCI) score (5.62 ± 2.81 vs. 4.89 ± 3.30, *p* = 0.1372) and the presence of malignancy (34.9% vs. 28.9%, *p* = 0.4092) were not significantly different between the two groups. Multiple liver abscesses were observed in nine patients who underwent EUS‐LAD (21%) and in 50 patients who underwent PTAD (21.3%) (*p* = 0.959). The mean diameter of the liver abscess was not significantly different between EUS‐LAD (7.79 ± 1.88 cm) and PTAD (7.84 ± 1.70 cm) (*p* = 0.084). Laboratory data such as white blood cell count, C‐reactive protein, bilirubin, aspartate aminotransferase, and alanine aminotransferase levels, were not significantly different between the two groups.

**TABLE 1:** Patients' characteristics.

	EUS‐guided drainage	Percutaneous drainage	*p*
Total number of patients, *n*	43	235	—
Median age, years (range)	71 (53–90)	76 (21–93)	0.129
Male:Female	25: 18	160: 75	0.184
Charlson comorbidity Index score, mean ± SD	5.62 ± 2.81	4.89 ± 3.30	0.137
Presence of malignancy, % (*n*)	34.9 (15)	28.9 (68)	0.409
Etiology, *n* (%)			
Biliary stricture	26 (60.5)	24 (10.2)	0.002
Bile duct stone	3 (7.0)	19 (8.1)	
Infection‐related	4 (9.3)	42 (17.9)	
Iatrogenic	4 (9.3)	9 (3.8)	
Others	2 (4.7)	5 (2.1)	
Idiopathic	18 (41.9)	137 (58.3)	
Multiple liver abscesses, % (*n*)	21.0 (9)	21.3 (50)	0.959
Diameter of liver abscess, cm, mean ± SD	7.79 ± 1.88	7.84 ± 1.70	0.084
WBC, /μL, mean ± SD	14140.0 ± 39736.4	7001.0 ± 3543.1	0.044
CRP, mg/L, mean ± SD	4.48 ± 5.35	2.71 ± 3.50	0.302
Bilirubin, mg/dL, mean ± SD	0.79 ± 0.51	0.69 ± 0.76	0.284
AST, U/L, mean ± SD	35.8 ± 31.2	34.1 ± 39.6	0.649
ALT, U/L, mean ± SD	28.3 ± 38.4	29.7 ± 29.9	0.914

Table 2 shows the results for pathogenic bacteria. In the EUS‐LAD group, *Escherichia coli* was the most common suspected pathogenic bacteria (25.6%, *n* = 11), followed by *Klebsiella pneumoniae* (21.0%, *n* = 9) and *Streptococcus anginosus* (9.3%, *n* = 4), and in the PTAD group, *Klebsiella pneumoniae* was the most common suspected pathogenic bacteria (31.9%, *n* = 75), followed by *Escherichia coli* (18.3%, *n* = 43), *Enterococcus faecium* (7.7%, *n* = 43), and *Streptococcus anginosus* (3.4%, *n* = 8). There were no significant differences in pathogenic bacteria between the two groups.

**TABLE 2:** Bacteriology results.

Bacterium identified, % (*n*)	EUS‐AD, *n* = 43	PTAD, *n* = 235	*p*
*Escherichia coli*	25.6 (11)	18.3 (43)	0.182
*Klebsiella pneumoniae*	21.0 (9)	31.9 (75)	0.104
*Enterococcus faecium*	1.6 (7)	7.7 (18)	0.070
*Streptococcus anginosus*	9.3 (4)	3.4 (8)	0.100
*Streptococcus conctellatus*	0 (0)	1.7 (4)	0.510
*Streptococcus intermedius*	0 (0)	1.2 (3)	0.603
*Candida glabrata*	7.0 (3)	3.0 (7)	0.190
*Streptococcus constelatus*	2.3 (1)	1.2 (3)	0.491
*Klebsiella aerogenes*	2.3 (1)	0.9 (2)	0.400
*Enterobacter cloacae*	2.3 (1)	0.9 (2)	0.400
*Entamoeba histolytica*	2.3 (1)	1.2 (3)	0.491
Not detected	9.3 (4)	14.0 (33)	0.290
Others	2.3 (1)	10.2 (24)	0.074

Table 3 shows the clinical outcomes of the PTAD and EUS‐LAD groups. Technical success of EUS‐LAD was obtained in almost all 43 patients; one patient failed EUS‐LAD because the needle could not reach the liver abscess. This patient underwent conservative treatment and was successfully treated. In the PTAD group, the technical success rate was 99.1% (233/235). Of the two failed patients, one patient died due to hemorrhagic shock caused by vessel injury during liver abscess puncture. In the other failed patient, during PTAD tube deployment, PTAD tube dislocation occurred due to respiratory fluctuation. This patient was successfully treated by fluid aspiration. Technical success was not significantly different between the two groups (*p* = 0.390). Mean procedure time was not significantly different between the EUS‐LAD (18.0 min) and PTAD (15.2 min) groups (*p* = 0.131). As the primary outcome, the clinical success rate was 97.7% (42/43) for EUS‐LAD and 91.5% (215/235) for PTAD, with no significant difference (*p* = 0.179). In the EUS‐LAD group, stent obstruction was not observed in any patients. Tube obstruction was observed in 12 patients (5.2%). However, regarding adverse events associated with the procedure, although notable adverse events were not observed in the EUS‐LAD group, various adverse events, such as peritonitis (*n* = 10) or PTAD tube dislocation (*n* = 5), were observed in the PTAD group (0% vs. 10.2%, *p* = 0.002). As noted above, procedure‐related mortality occurred in one patient in the PTAD group. Although there was no significant difference in length of hospital stay between the two groups, the discharge rate within 28 days was significantly higher in the EUS‐LAD group (79.1%, 34/43) than in the PTAD group (58.3%, 137/235). During follow‐up, the liver abscess recurrence rate was not significantly different between the EUS‐LAD (4.7%, 2/43) and PTAD groups (5%, 12/235) (*p* = 0.900). The drainage target area was significantly different between the two groups; therefore, a propensity score‐matched analysis using several factors such as sex, age, and drainage target area was performed. Table 4 shows the clinical outcomes of the two groups after propensity score‐matched analysis. The clinical success rate was significantly higher in the EUS‐LAD group (97.7%, 42/43) than in the PTAD group (79.1%, 34/43) (*p* = 0.007). However, one of the advantages of EUS‐LAD might be the ability to deploy a metal stent; therefore, a propensity score‐matched analysis including patients who underwent EUS‐LAD using a SEMS was performed. As shown in Table 5, 25 patients were included in each group. The clinical success rate was significantly higher in the EUS‐LAD group (100%, 25/25) than in the PTAD group (84%, 21/25) (*p* = 0.037). Adverse events were also significantly higher in the PTAD group (16%, 5/25) than in the EUS‐LAD group (*p* = 0.025). In addition, the median length of hospital stay was significantly shorter in the EUS‐LAD group (15 days) than in the PTAD group (22 days) (*p* = 0.005).

**TABLE 3:** Clinical outcomes.

	EUS‐AD	PTAD	*p*
Total number of patients, *n*	43	235	—
Drainage target area, *n* (%)			
Left	29 (67.4)	89 (37.9)	< 0.001
Right	14 (32.6)	146 (62.1)	
Kind of drainage device, *n* (%)			
Metal stent	25 (58.1)	0 (0)	< 0.001
Plastic stent	12 (28.0)	0 (0)	
External tube	6 (13.9)	235 (100)	
Technical success rate, % (*n*)	97.7 (42)	99.1 (233)	0.390
Procedure time, min, mean ± SD	18.0 ± 13.8	15.2 ± 10.2	0.131
Clinical success rate, % (*n*)	97.7 (42)	91.5 (215)	0.179
Adverse event, *n* (%)	0 (0)	24 (10.2)	0.002
Tube dislocation	0 (0)	5 (2.1)	
Self‐tube removal	0 (0)	1 (0.4)	
Peritonitis	0 (0)	10 (4.3)	
Bleeding	0 (0)	2 (0.9)	
Empyema	0 (0)	3 (1.3)	
Pneumothorax	0 (0)	1 (0.4)	
Pleural effusion	0 (0)	2 (0.9)	
Length of hospital stay, days, median [IQR]	18.0 [12.0, 25.5]	26.0 [17.0, 36.5]	0.211
Discharge rate within 28 days, % (*n*)	79.1 (34)	58.3 (137)	0.010
Mortality rate within 28 days, % (*n*)	2.3 (1)	5.1 (12)	0.485
Follow‐up period, days, median [IQR]	141.0 [56.5, 502.5]	161.0 [54.0, 575.5]	0.446
Recurrence rate of liver abscess, % (*n*)	4.7 (2)	5 (12)	0.900

**TABLE 4:** Clinical outcomes of EUS‐AD and PTAD after propensity score matching.

	EUS‐AD	PTAD	*p*
Total number of patients, *n*	43	43	—
Median age, years (range)	71 (36–90)	70 (21–90)	0.890
Male:Female	25:18	27:16	0.659
Drainage target area, *n* (%)			
Left	29 (67.4)	29 (67.4)	1.00
Right	14 (32.6)	14 (32.6)	
Kind of drainage device, *n* (%)			
Metal stent	25 (58.1)	0 (0)	< 0.001
Plastic stent	12 (28.0)	0 (0)	
Tube	6 (13.9)	43 (100)	
Technical success rate, % (*n*)	100 (43)	100 (43)	1.00
Procedure time, min, mean ± SD	18.0 ± 13.8	15.1 ± 11.7	0.216
Clinical success rate, % (*n*)	97.7 (42)	79.1 (34)	0.007
Adverse events, % (*n*)	0 (0)	4.7 (2)	0.247
Tube dislocation	0 (0)	2.3 (1)	
Peritonitis	0 (0)	2.3 (1)	
Length of hospital stay, days, median [IQR]	18.0 [12.0, 25.5]	23.0 [16.0, 34.5]	0.113
Mortality rate within 28 days, % (*n*)	0 (0)	7.0 (3)	0.078
Follow‐up period, days, median [IQR]	141.0 [56.5, 502.5]	150.0 [41.5, 532.0]	0.656
Recurrence of liver abscess, % (*n*)	4.7 (2)	2.3 (1)	0.557

**TABLE 5:** Clinical outcomes of EUS‐AD with MS versus PTAD after propensity score matching.

	EUS‐AD with MS	PTAD	*p*
Total number of patients, *n*	25	25	—
Median age, years (range)	67 (36–90)	71 (33–88)	0.28
Male:female	18:07	14:11	0.239
Drainage target area, *n* (%)			
Left	15 (60)	18 (72)	0.37
Right	10 (40)	7 (28)	
Technical success rate, % (*n*)	100 (25)	100 (25)	1
Kind of drainage device, *n* (%)			
Metal stent	25 (100)	0 (0)	< 0.001
Plastic stent	0 (0)	0 (0)	
Tube	0 (0)	25 (100)	
Procedure time, min, mean ± SD	11.2 ± 8.34	17.2 ± 12.8	0.149
Clinical success rate, % (*n*)	100 (25)	84.0 (21)	0.037
Adverse events, % (n)	0 (0)	20 (5)	0.025
Tube dislocation	0 (0)	4 (1)	
Peritonitis	0 (0)	16 (4)	
Length of hospital stay, median [IQR]	15.0 [12.0, 20.0]	22.0 [16.0, 32.0]	0.005
Mortality rate within 28 days, % (*n*)	0 (0)	4.0 (1)	0.312
Follow‐up period, median [IQR]	200.0 [39.0, 716.0]	312.0 [51.0, 881.0]	0.614
Recurrence of liver abscess, % (*n*)	0 (0)	4.0 (1)	0.312

## 4 Discussion

According to a recent meta‐analysis of percutaneous transhepatic needle aspiration (PNA) and PTAD including 15 randomized, controlled trials involving 1626 patients [13], the technical success rate of PTAD was 96.7% (758/784). In addition, according to several recent randomized trials [14, 15], the technical success rate of PTAD was around 95%. In the present study, the technical success of PTAD was achieved in almost all cases (99.1%, 233/235); therefore, the present study might be reliable, although many expert endoscopists were included. In the present study, the clinical success rate was significantly higher in the EUS‐LAD group than in the PTAD group in the propensity score‐matched analysis. Moreover, in the case of EUS‐LAD using a SEMS, significant differences were observed in not only clinical success but also adverse events and length of hospital stay. This can be explained by several reasons associated with the advantages of EUS‐guided access compared with percutaneous transhepatic access. First, the resolution of ultrasound images and sensitivity of color Doppler images might be higher in EUS than in AUS. Although this may depend on the location of the liver abscess, a liver abscess can be identified more closely with EUS than with AUS. In addition, small vessel injury can be easily avoided in EUS because the puncture needle can be adjusted using the elevator of EUS. Therefore, bleeding as a complication is theoretically less likely in EUS. Indeed, severe bleeding occurred in the PTAD group, but no bleeding was observed in the EUS group. Second, the drainage device is larger in EUS than in PTAD. The pus of the liver abscess can obstruct the drainage device if it is too thin, which may decrease the drainage effect. In contrast, if EUS‐guided access is selected, a SEMS with a diameter of 8 mm or 10 mm can be used. Therefore, the pus of the liver abscess can be more easily drained than with PTAD. In addition, a SEMS has a self‐expansion force, which may produce a tamponade effect; therefore, leakage of fluid contents into the abdominal cavity along the drainage device is much less likely. Thus, clinical success or length of hospital stay or adverse events can be better in the EUS‐LAD group. Third, because of internal drainage in EUS‐LAD, PTAD tube complications such as PTAD tube removal did not occur.

To date, several studies of the technical feasibility of EUS‐LAD and only a few studies comparing PTAD and EUS‐LAD have been reported [6, 7, 14, 15, 16, 17]. According to a meta‐analysis of EUS‐LAD [18], a pooled analysis of data from these studies showed that EUS‐LAD had high technical success (90.7%) and clinical success rates (90.7%). Therefore, EUS‐LAD itself might be feasible and effective. Shahid et al. conducted a comparison study of EUS‐LAD and PTAD in a retrospective, multicenter setting [7]. Their study included 74 patients, of whom 30 underwent EUS‐LAD and 44 underwent PTAD. The median abscess size was 8.45 × 6 cm^2^ in the EUS‐LAD group versus 7.3 × 5.5 cm^2^ in the PTAD group. All of the abscesses in the EUS group were left‐sided, whereas the PTAD group contained both left‐ and right‐sided abscesses (29 and 15, respectively). Technical success was 100% in both groups. The duration to resolution of symptoms from the initial procedure was significantly shorter (10.9 days less, *p* < 0.00001) in the EUS‐LAD group than in the PTAD group. The length of hospital stay was also shorter in the EUS‐LAD group by 5.2 days (*p* = 0.000126). In addition, the PTAD group had a significantly higher number of adverse events (*n* = 27 [61%]) than the EUS group (*n* = 5 [17%]; *p* = 0.0001). Therefore, they concluded that EUS‐guided drainage is an efficacious and safe intervention for the management of liver abscesses. However, as they noted in their study, the study had a limitation, such as the inclusion of only left liver abscess cases. In contrast, in the present study, right liver abscess cases were included, with about 35% (15/43) in the EUS‐LAD group. Moreover, to reduce the heterogeneity of the study population, a propensity score‐matched analysis using the drainage site was performed. After this analysis, EUS‐LAD had a higher clinical success rate than PTAD. When an SEMS was used, clinical outcomes such as adverse events and length of hospital stay were better with EUS‐LAD than with PTAD. To the best of our knowledge, the present study may be the first study comparing EUS‐LAD and PTAD including right liver abscess cases using a propensity score‐matched analysis. However, EUS‐LAD itself has several limitations. First, although EUS‐LAD for left liver abscesses can be performed in most cases, in right liver abscess cases, if the liver abscess is far from the duodenum, detection of the liver abscess might be challenging. In addition, the needle reaching into the liver abscess may also be difficult. Indeed, in the present study, the needle could not reach into the liver abscess because the right liver abscess was far from the duodenum. For successful performance of EUS‐LAD in the right hepatic lobe, several specific anatomical conditions should be satisfied. The distance between the liver abscess and the duodenum should ideally be within 8 cm. If this distance exceeds 8 cm, the puncture needle may not reach the abscess, thereby precluding completion of the procedure. Similarly, abscesses located in segments 7 and 8 are often situated at a greater distance, and in cases where the abscess diameter is ≤ 5 cm, adequate visualization may be technically challenging. Second, in cases of surgically altered anatomy or duodenal obstruction, EUS‐LAD is not indicated for a right liver abscess because it may be impossible to identify the right liver.

Regarding stent selection during EUS‐LAD, we generally select PCSEMS. The reason is that, after EUS‐LAD, once the cavity of the liver abscess is decompressed, the stent may be displaced from the abscess by the lateral wall, potentially resulting in stent migration into the abdominal cavity. On the other hand, if the distal end of the SEMS is in contact with the abscess wall after EUS‐LAD, mucosal hyperplasia may readily occur. Therefore, in such situations, FCSEMS may be considered as an alternative. In the presented study, stent removal could be successfully performed without any adverse events; however, if the uncovered part was placed in hepatic parenchyma, mucosal hyperplasia can be easily complicated. As a result, stent removal might be challenging; therefore, stent selection should be carefully attempted.

The present study also has several limitations. First, although a relatively large number of liver abscess cases was included, cases of EUS‐LAD, especially for right liver abscesses, were few. Second, the present study was retrospective in nature, but a propensity score‐matched analysis was used to decrease the effect of this limitation. Third, the technical success rate of PTAD might be lower compared with a previous report because some patients underwent PTAD by endoscopists. Therefore, a randomized, controlled trial comparing EUS‐LAD and PTAD by experienced radiologists would be needed to verify the present results.

In conclusion, EUS‐LAD using a SEMS might be one of the options, but further randomized, controlled trials are needed.

## Author Contributions

Takeshi Ogura wrote the paper. Taira Kuroda, Takanori Matsuura, Jun Kitadai, Koh Kitagawa, Masahiro Itonaga, Kotaro Takeshita, Tomoaki Matsumori, Tomoya Emori, Mamoru Takenaka, Hajime Imai, Koichiro Mandai, Shuhei Shintani, Nao Fujimori, Hideyuki Shiomi, Masanori Asada, Ryota Sagami, Hirotsugu Maruyama, Tsukasa Ikeura, Masaaki Shimatani, Hidefumi Nishikiori, Kazuyuki Matsumoto, Masahito Kokubu, Hideki Kamada, Yusuke Ishida, Akitoshi Hakoda, and Masayuki Kitano played roles in data interpretation, revised the work critically for important intellectual content, provided final approval of the version to be published, and agreed to be accountable for all aspects of the work in ensuring that questions related to the accuracy or integrity of any part of the work are appropriately investigated and resolved.

## Funding

The authors have nothing to report.

## Ethics Statement

A priori approval for this study was given by the Human Research Committee of Osaka Medical and Pharmaceutical University (IRB No. 2024‐250).

## Consent

Obtained by optout.

## Conflicts of Interest

Masayuki Kitano receives grants from Sumitomo Bakelite Co. Ltd. Masayuki Kitano is editor‐in‐chief of *Digestive Endoscopy*. Kouske Minaga and Mamoru Takenaka are associate editors of *Digestive Endoscopy*. Other authors have no conflicts of interest to declare.
